# Exploring the link between toxic metal exposure and ADHD: a systematic review of pb and hg

**DOI:** 10.1186/s11689-024-09555-8

**Published:** 2024-08-01

**Authors:** Reyhane Farmani, Omid Mehrpour, Alireza Kooshki, Samaneh Nakhaee

**Affiliations:** 1 Student Research Committee, Birjand University of Medical Sciences, Birjand, Iran; 2 Michigan Poison & Drug Information Center, Wayne State University School of Medicine, Detroit, MI USA; 3 Medical Toxicology and Drug Abuse Research Center (MTDRC), Birjand University of Medical Sciences, Birjand, Iran

**Keywords:** Attention-deficit/hyperactivity disorder, ADHD, Heavy metals, Lead, Pb, Mercury, Hg, Neurodevelopmental disorders

## Abstract

**Introduction:**

Attention-Deficit/Hyperactivity Disorder (ADHD) is a recognized neurodevelopmental disorder with a complex, multifactorial origin. Lead (Pb) and mercury (Hg) are highly toxic substances that can potentially impair brain development and have been implicated in the development of ADHD. This systematic review aims to analyze the epidemiological literature regarding the association between Pb and Hg exposure and the diagnosis of ADHD.

**Methods:**

From November 1983 to June 2, 2023, a comprehensive search was conducted in multiple databases and search engines, including PubMed, Web of Science, Scopus, and Google Scholar. Observational studies (case-control, cohort, and cross-sectional) measuring Pb and Hg levels in various biological samples (blood, hair, urine, nail, saliva, teeth, and bone) of children with ADHD or their parents and their association with ADHD symptoms were included.

**Results:**

Out of 2059 studies, 87 met the inclusion criteria and were included in this systematic review. Approximately two-thirds of the 74 studies investigating Pb levels in different biological samples reported associations with at least one subtype of ADHD. However, most studies examining Hg levels in various biological samples found no significant association with any ADHD subtype, although there were variations in exposure periods and diagnostic criteria.

**Conclusion:**

The evidence gathered from the included studies supports an association between Pb exposure and the diagnosis of ADHD, while no significant association was found with Hg exposure. Importantly, even low levels of Pb were found to elevate the risk of ADHD. Further research is needed to explore the comprehensive range of risk factors for ADHD in children, considering its significance as a neurodevelopmental disorder.

## Introduction

Attention-Deficit/Hyperactivity Disorder (ADHD) is a well-known neurodevelopmental disorder characterized by symptoms of inattention, impulsivity, and hyperactivity, resulting in significant functional impairment [1]. The condition is particularly important due to its early childhood onset and persistence into adulthood [2]. Children diagnosed with ADHD often struggle with task focus, learning difficulties, and weakened interpersonal skills, leading to self-confidence issues and negative emotional states. Consequently, their personal, academic, and social performance is adversely affected [3].

Globally, ADHD affects approximately 5% of children and adolescents, with an increasing trend observed in recent years [1]. In the United States, the prevalence of diagnosed ADHD cases among children and adolescents has risen from 6.1% in 1998 to 10.2% in 2016 [4]. Furthermore, ADHD is also a concern in adulthood, with persistent cases from childhood and newly symptomatic cases estimated to affect 2.58% and 6.76% of the adult population, respectively [5].

Considering the escalating prevalence of ADHD, it is crucial to explore environmental factors that may contribute to its development. Among these factors, certain metals, known for their neurotoxic effects, have gained attention [6]. Human exposure to these metals can occur through various sources such as industrial sites, soil and air pollution, and dietary intake [7].

Lead (Pb) is a highly dangerous substance, ranked second in terms of hazardousness by the Agency for Toxic Substances and Disease Registry (ATSDR) [8]. Various industrial processes, such as lead ore mining and smelting, pottery production, utilization of lead-lined food and drink containers, lead-based painting, and battery recycling, can result in lead exposure [9, 10]. Even at low concentrations, lead can impair brain development and adversely impact neurobehavioral functions long-term, resulting in poor academic performance and diminished intelligence quotient [11]. Several scientific studies have implicated it as a prevalent risk factor contributing to the development of ADHD in children [12–14]. Additionally, there is evidence indicating that lead can traverse the placenta during pregnancy, and elevated prenatal lead levels are associated with deceleration in sensorimotor or visual-motor development in children [15, 16]. Lead is also responsible for structural alterations in neurons, synaptogenesis, myelination, and neuron differentiation [17]. Studies indicate that lead alters neurogenesis and affects cortical neurons, ultimately leading to cognitive disabilities [18]. Traffic continues to be a concern regarding atmospheric lead pollution [19].

The central nervous system is the primary target of lead exposure, especially during developmental stages, due to its ability to readily cross the blood-brain barrier [17]. Multiple factors undoubtedly influence the neurotoxicity associated with lead exposure; however, the impacts of lead on the brain can be divided into morphological or pharmacological effects. Morphological effects involve structural alterations in brain cells, influencing crucial processes such as synaptogenesis, myelination, and neuron differentiation. Meanwhile, pharmacological effects involve ion mimicry, wherein Pb^2+^ competes with essential ions for their functional roles and insertion sites. As a result, Pb^2+^ is incorporated into the brain, disrupting synaptic neurotransmission, causing mitochondrial dysfunction, and potentially inducing neuroinflammation. Consequently, these mechanisms are responsible for lead intoxication’s neurotoxic effects on the neurobehavioral system [8].

Mercury (Hg) is ranked third in terms of hazardousness, according to ATSDR. The significance of mercury toxicity is not surprising, given the diverse routes of human exposure, such as fish consumption, dental amalgam fillings, and the utilization of mercury-based preservatives like thimerosal (ethylmercury thiosalicylate.) in vaccinations [20, 21]. Due to its ability to cross the placenta and blood-brain barrier, mercury poses a significant risk of neurotoxicity. Notably, the developing brain is particularly vulnerable to these effects, potentially leading to long-lasting consequences [22]. Evidence suggests a potential association between both prenatal and postnatal exposure to mercury and the manifestation of neurodevelopmental complications, including ADHD, diminished cognitive abilities (low IQ), and language impairments [23, 24]. This toxic element inhibits the sulfhydryl-containing enzymes and increases the lipid peroxidation and reactive oxygen species (ROS) levels. Hg is widely discussed for its effect on brain cells through oxidative stress and apoptotic processes [25].

The previous studies emphasize the significance of lead exposure as a potential contributing factor to the development of ADHD. In 2019, a systematic review study [26] was conducted to examine the literature on the impact of lead exposure on children diagnosed with ADHD. This review specifically focused on studies conducted between July 1, 2013, and June 30, 2018. Their findings revealed a significant association between lead exposure and ADHD in 12 out of the 17 studies reviewed [26]. A recent systematic review comprising 31 papers examined the impact of mercury (Hg) on ADHD. The study concluded that the available information regarding the effects of mercury on ADHD is limited [27].

To our knowledge, two similar studies, each with limitations, have been conducted on these toxic and widespread metals.

Previous studies on this matter have been limited to one metal, and we tend to evaluate the effect of two of the most common toxic metals (Pb and Hg) on ADHD. The year of study has also been expanded in our research. We comprehensively reviewed these metals in all available human body samples to better understand their role in ADHD. This systematic review aims to thoroughly evaluate the available evidence on the association between two specific toxic metals, lead (Pb) and mercury (Hg), in various biological specimens (blood, hair, urine, teeth, nails, and bone) and ADHD.

## Methods

### Design and search strategy

This systematic review study adhered to the guidelines outlined in the Preferred Reporting Items for Systematic Reviews and Meta-Analyses (PRISMA). A search was conducted in four databases/search engines: PubMed, Scopus, Web of Science, and Google Scholar until June 2, 2023. No restrictions were imposed on the publication dates, and all available studies from the earliest records were considered. To capture relevant studies, we utilized keywords and medical subject headings (MeSH) terms to search for the titles or abstracts of the studies. The search strategies employed in each database are summarized in Table 1. Endnote software was used to facilitate data extraction and management from the databases.

**Table 1:** Search strategies in different databases for retrieving the relevant documents

Database/ search engine	Search strategy	Results
Pub Med	(((((((((((((((“Attention Deficit-Hyperactivity Disorder*“[Title/Abstract]) OR (“Attention Deficit Disorder*“[Title/Abstract])) OR (“Attention-Deficit/Hyperactivity Disorder*“[Title/Abstract])) OR (“Attention Deficit Hyperactivity Disorder*“[Title/Abstract])) OR (“Attention Deficit Disorders with Hyperactivity“[Title/Abstract])) OR (“Attention Deficit Disorder with Hyperactivity“[Title/Abstract])) OR (ADHD[Title/Abstract])) OR (ADDH[Title/Abstract])) OR (“Attention-Deficient Hyperactivity Disorder“[Title/Abstract])) OR (“Neurodevelopmental Disorder*“[Title/Abstract])) OR (“Neurodevelopmental Disease*“[Title/Abstract])) OR (Impulsivity[Title/Abstract])) OR (Inattention[Title/Abstract])) OR (ADHD[MeSH Terms])) OR (“Attention Deficit Disorder with Hyperactivity“[MeSH Terms])) AND (((((((((((((Lead[MeSH Terms]) OR (Pb[Title/Abstract])) OR (Mercury[Title/Abstract])) OR (Hg[Title/Abstract])) OR (“Toxic Metal*“[Title/Abstract])) OR (“Heavy Metal*“[Title/Abstract])) OR (“Trace Metal*“[Title/Abstract])) OR (“Methylmercury“[Title/Abstract])) OR (“Environmental Toxicant“[Title/Abstract])) OR (“Chemical Hazard“[Title/Abstract])) OR (“Thimerosal“[Title/Abstract])) OR (Mercury[MeSH Terms])) OR (“Metals, Heavy“[MeSH Terms]))	619
Scopus	( TITLE-ABS ( “Attention Deficit-Hyperactivity Disorder*” OR “Attention Deficit Disorder*” OR “Attention-Deficit/Hyperactivity Disorder*” OR “Attention Deficit Hyperactivity Disorder*” OR “Attention Deficit Disorders with Hyperactivity” OR “Attention Deficit Disorder with Hyperactivity” OR ADHD OR ADDH OR “Attention-Deficient Hyperactivity Disorder” OR “Neurodevelopmental Disorder*” OR “Neurodevelopmental Disease*” OR impulsivity OR inattention ) AND ( ( CHEMNAME (lead) ) OR TITLE-ABS (pb OR mercury OR hg OR “Toxic Metal*” OR “Heavy Metal*” OR “Trace Metal*” OR “Methylmercury” OR “Environmental Toxicant” OR “Chemical Hazard” OR “Thimerosal” ) ) )	517
Web of Science	(AB=(“Attention Deficit-Hyperactivity Disorder*” OR “Attention Deficit Disorder*” OR “Attention-Deficit/Hyperactivity Disorder*” OR “Attention Deficit Hyperactivity Disorder*” OR “Attention Deficit Disorders with Hyperactivity” OR “Attention Deficit Disorder with Hyperactivity” OR ADHD OR ADDH OR “Attention-Deficient Hyperactivity Disorder” OR “Neurodevelopmental Disorder*” OR “Neurodevelopmental Disease*” OR Impulsivity OR Inattention)) AND (TI=(Lead) OR AB=(Pb OR Hg OR Mercury OR “Toxic Metal*” OR “Heavy Metal*” OR “Trace Metal*” OR “Methylmercury” OR “Environmental Toxicant” OR “Chemical Hazard” OR “Thimerosal”))	567
	TI=((“Attention Deficit-Hyperactivity Disorder*” OR “Attention Deficit Disorder*” OR “Attention-Deficit/Hyperactivity Disorder*” OR “Attention Deficit Hyperactivity Disorder*” OR “Attention Deficit Disorders with Hyperactivity” OR “Attention Deficit Disorder with Hyperactivity” OR ADHD OR ADDH OR “Attention-Deficient Hyperactivity Disorder” OR “Neurodevelopmental Disorder*” OR “Neurodevelopmental Disease*” OR Impulsivity OR Inattention) AND (Lead OR Pb OR Hg OR Mercury OR “Toxic Metal*” OR “Heavy Metal*” OR “Trace Metal*” OR “Methylmercury” OR “Environmental Toxicant” OR “Chemical Hazard” OR “Thimerosal”))	147
Google Scholar	allintitle: (“Attention Deficit Hyperactivity Disorder*” OR ADHD OR impulsivity OR “Attention Deficit Disorder*” OR inattention OR “neurodevelopmental disorder*”)(lead OR mercury OR “Heavy metal*” OR Thimerosal OR Pb OR Methylmercury OR Hg)	209

### Eligibility criteria for study selection

#### Inclusion criteria

Human observational studies (case-control, cohort, cross-sectional) that assessed the relationship of ADHD with at least one of the heavy metals of interest, namely lead (Pb) or mercury (Hg), were included in this systematic review. The age restriction for ADHD subjects was set to encompass individuals up to 20 years old, as the review specifically examined the association of heavy metals and attention-deficit/hyperactivity disorder (ADHD) in children. No language or time limitations were imposed, and articles written in English or those with at least one English abstract were considered. Additionally, the reference lists of the included studies were screened for relevant publications.

#### Exclusion criteria

Experimental research, books, review articles, or letters to the editor were excluded from this systematic review. Studies that did not report relevant results were also excluded at each stage of the document screening process. Initially, the records retrieved from the databases were integrated, and duplicate records were removed. Subsequently, articles were screened based on their titles and abstracts, excluding those not meeting the inclusion criteria. Finally, the full texts of the remaining articles were thoroughly reviewed.

### Data extraction

Relevant data from the included studies were extracted and organized. An electronic data abstraction form was used to document various study characteristics, including the first author’s name, publication year, country where the study was conducted, research design, number of participants, age range, gender distribution, criteria used to diagnose ADHD, specific ADHD symptoms evaluated, and key study results.

## Results

**Fig. 1:**
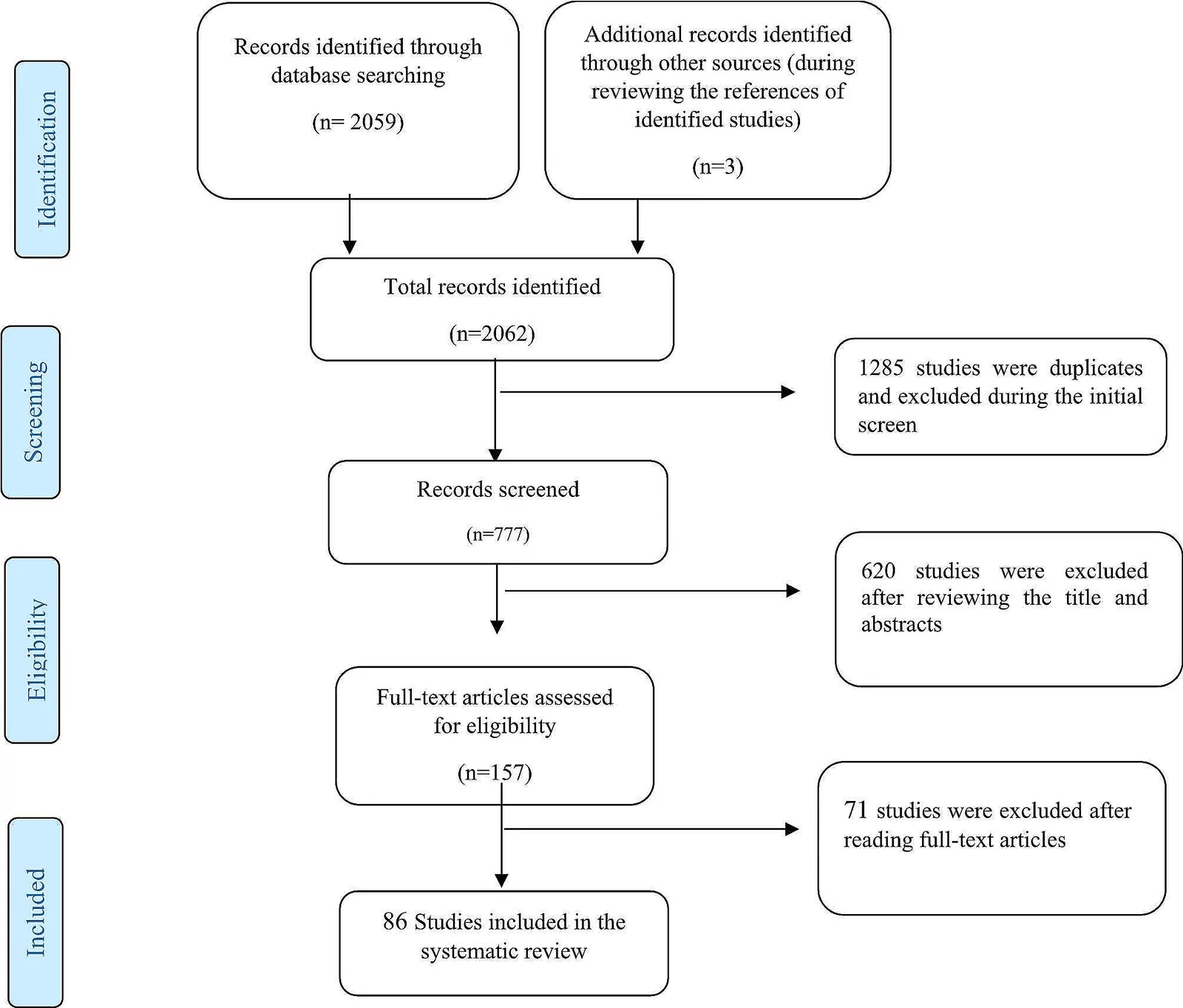
PRISMA Flowchart of the literature search and strategy for selecting relevant documents

From the initial search across various databases and search engines, 2059 studies were identified. After removing duplicates using Endnote Software, 1209 unique studies remained. Applying the pre-defined study inclusion criteria to the titles and abstracts resulted in 120 relevant articles for further examination. Following a thorough assessment of the full texts, 86 articles were included in this systematic review (Fig. 1).

The selected studies investigated the measurement of at least one of the metals of interest, lead (Pb) or mercury (Hg), in various biological samples, such as blood, hair, urine, saliva, teeth, or bones, obtained from children diagnosed with ADHD or their parents. Among the selected studies (*n* = 86), there were 35 case-control studies, 26 cohort studies, and 25 cross-sectional studies. Metal concentrations were predominantly measured in children (66 studies), while ten studies focused on mothers and another ten on mothers and children. In terms of age ranges for ADHD cases, the youngest subject was one year old, while the oldest was 20 years old.

The publication years of the included studies range from 1983 to 2023.

The assessment of heavy metal concentrations primarily utilized techniques such as inductively coupled plasma (ICP) or atomic absorption spectrometry (AAS), although some studies employed methods such as Direct Mercury Analyzer [28–30], Anodic Stripping Voltammetry [31, 32], Fluorescence Spectrometry [33, 34], Gas Chromatography and High-resolution Mass Spectrometry [35], or K-shell X-ray Fluorescence [36]. Various questionnaires were employed for the diagnosis of ADHD with determining symptoms (inattention, hyperactivity/impulsivity, or combined), largely based on criteria outlined in the Diagnostic and Statistical Manual of Mental Disorders (DSM) and the International Classification of Diseases (ICD). Most studies were conducted in China, South Korea, and the United States. Tables 2 and 3 demonstrate the findings of these studies.

**Table 2:** Characteristics of included studies for assessments of the relationship between lead concentrations in different biological samples and ADHD

First author (year)	Country	Type of Study	Sample size (control, case)	Mean age (control, case)	Gender F/M (control, case)	ADHD Symptoms Measured	Diagnostic criteria for ADHD	Assessment method	Biomarker	Main result
Amgalan B 2020 [83]	Mongolia	Case-control	30, 30	7 to 12 9.34, 9.14	6/24, 6/24	Combined ADHD, Inattention, Hyperactivity/Impulsivity	DSM-IV	ICP-MS	Hair	Pb ↑ (PI/PH/PC) Hair Pb levels were significantly higher in all three subtypes of ADHD
Al-Ayadhi L 2005 [88]	Saudi Arabia	Case-control	80, 8	Up to 14 7.2, 6	NM, 0/8	Inattention	E-2	AAS	Hair	Pb ↑ (PI) Children with ADD exhibited significantly elevated Pb concentrations in their hair samples
Arbuckle T 2016 [12]	Canada	Cohort	1031, 49	6 to 11	NM	Combined ADHD, Inattention	SDQ	ICP-MS	Blood	Pb ↑ (PI/PC) An increase of 1 unit in ln-blood Pb was associated with 2.08-fold higher odds of ADD/ADHD
Awaga M 2020 [50]	Egypt	Case-control	30, 70	4 to 12 6.70, 6.52	5/25, 12/58	Combined ADHD, Hyperactivity/Impulsivity, Inattention	DSM-IV	AAS	Blood	Pb ↑ (PC) Pb ↔ (PI/PH) There was a strong association between BLLs and the combined ADHD group
Barg G 2018 [72]	Uruguay	Cross-sectional	206	5 to 8 6.7	83/123	Hyperactivity, Inattention, Combined ADHD	CTRS-R,	AAS	Blood	Pb ↔ (PI/PH/PC)
Braun J 2006 [64]	USA	Cross-sectional	4704 4.2% ADHD	4 to 15	NM	Combined ADHD	Parents reported	GFAAS	Blood	Pb ↑ (PC) A significant dose-response relationship was found between Pb exposure and ADHD. The highest BLLs (2.0–5 µg/dL) are associated with 4.5-fold higher ADHD risk compared to the lowest quintile (nondetectable to 0.7 µg/dL)
Boucher O 2012 [49]	Canada	Cohort	279	11.3	141/138	Inattention, Hyperactivity/Impulsivity	DBD (Teachers reported) (DSM-IV)	ICP-MS	Blood	Pb ↑ (PH) Pb ↔ (PI)
Chan T 2015 [95]	USA	Cross-sectional	262	11 to 13	128/138	ADHD, Inattention, Hyperactivity/Impulsivity	DBDRS	ICP-OES	Teeth	Pb ↔ (PI/PH/PC) No association between BLLs and ADHD was found after adjusting
Chiodo L 2007 [119]	USA	Cohort	506	7 6.9	249/257	Hyperactivity/Impulsivity, Inattention	CPT, CTRS-39,TRF	AAS	Blood	Pb ↑ (PI/PH) Mean BLLs were associated with increased inattentive behavior and hyperactivity but not impulsivity
Cho S 2010 [37]	South Korea	Cross-sectional	639	8 to 11 9.05	306/333	Inattention, Hyperactivity	K-ARS (DSM) (Parents and teachers reported)	AAS	Blood	Pb (T reported) ↑ (PI/PH) Pb (P reported) ↔ (PI/PH) Teacher-rated ADHD symptoms were significantly positively associated with BLLs, whereas parent-rated ADHD symptoms did not show a significant association with BLLs
Choi J 2020 [67]	South Korea	Case-control	96,259	5 to 18 10.5, 8.8	44/52, 57/202	Inattention, Hyperactivity/Impulsivity, Combined ADHD	K-ARS (DSM)	GFAAS	Blood	Pb ↔ (PI/PH/PC) No significant difference in BLLs was observed between the ADHD and HC groups after adjusting for age, sex, and IQ
Choi W 2016 [51]	South Korea	Cohort	2052, 107	7 to 9 years	1126/1033, 34/73	Combined ADHD, Inattention, Impulsivity	K-ARS (DSM)	AAS	Blood	Pb↑ (PC) Children with BLLs above 2.17 µg/dL (highest quartile) had a relative risk of 1.552 (95% CI) for ADHD symptoms compared to those with BLLs below 2.17 µg/dL
Darougar S 2012 [77]	Iran	Case-control	100, 100	NM	41/59, 24/76	Combined ADHD	CBRS	AAS	Blood	Pb ↔ (PC)
Desrochers-Couture M 2019 [76]	Canada	Cohort	212	9–14 (11.34) (Childhood) 16–22 (18.47) (adolescent)	118/94 (adolescent)	Hyperactivity/Impulsivity	DBD, BAARS (Teachers reported) (DSM-IV)	NA	Cord Blood Childhood Blood Adolescence Blood	Pb ↔ (PH)
Dikme G 2013 [78]	Turkey	Case-control	59,17	1.6 to 16	NM	Combined ADHD	DSM-IV	AAS	Blood	Pb ↔ (PC)
El-Morsi 2019 [87]	Egypt	Case-control	48,54	5 to 14 7.83, 8.09	29/19, 34/20	Combined ADHD, Inattention, Hyperactivity	DSM-IV	ICP	Hair	Pb ↑ (PC) A significant difference was observed in Pb levels between the ADHD and control groups, with higher risks observed in female patients with the inattention type
Ethier A 2015 [73]	Canada	Cohort	27	8.6 to 12.6 11.2	9/18	Inattention, Impulsivity	Classic Posner paradigm	GFAAS	Blood	Pb ↔ (PI/PH)
									Cord blood	Pb ↑ (PI/PH) Pb is associated with more inattention and impulsivity
Firouzkouhi Moghaddam M 2015 [52]	Iran	Case-control	23, 20	4 to 12	12/11, 10/10	Combined ADHD	DSM-IV	GFAAS	Blood	Pb ↑ (PC) ADHD children demonstrated significantly higher Pb levels compared to the control group
Fergusson D 1988 [94]	New Zealand	Cohort	888	8 or 9	NM	Inattention, Hyperactivity/Impulsivity	Rutter and Conner’s behavior scale rating	NM	Blood	Pb ↑ (PH) Pb ↔ (PI) Blood Pb concentration correlated with impulsivity, but not with attention, in both prenatal and current measurements
Forns J 2013 [98]	Spain	Cohort	385	4.43	186/199	Inattention, Hyperactivity/Impulsivity	DSM-IV	Q-ICP-MS	Maternal urine during pregnancy	Pb ↔ (PI/PH)
Fraser S 2006 [47]	Canada	Cohort	101	5.4 30.2	NM	Inattention, Hyperactivity/Impulsivity	IBR	GFAAS	Cord blood	ND
Froehlich T 2009 [65]	USA	Cross-sectional	2588	8 to 15	NM	Combined ADHD	DSM-IV	GFAAS	Blood	Pb ↑ (PC) Children in the highest tertile of Pb levels (> 1.3-5 µg/dL) had over a two-fold increased risk of ADHD (adjusted odds ratio [aOR] for third versus first tertile: 2.3)
Gari M 2022 [23]	Poland	Cohort	436	Parental age: 29.3	NM	Hyperactivity, Inattention	SDQ	ICP-MS	cord blood	Pb ↔ (PI/PH)
Geiere D 2017 [38]	USA	Cross-sectional	29805403, 2956755	10 to19 14.52	NM	Inattention	NHANES dataset	ICP-MS	Blood	Pb ↑ (PI) The prevalence ratio of ADD was significantly higher (1.59) in the 75th to 100th percentile group compared to the reference group (0 to 50th percentile)
Gittelman R 1983 [93]	USA	Cross-sectional	33, 103	10.4, 10.0	16/17, 11/92	Hyperactivity	Teacher Rating Scale, Psychiatrist	NM	Urine	Pb ↔ (PH) 58% of hyperactive children and 39% of normal developing children had Pb levels exceeding 0.08 mg/L
Ha M 2009 [53]	South Korea	Cohort	1663, 115	6 to 10	NM	Combined ADHD	CPRS	AAS	Blood	Pb ↑ (PC) The prevalence of ADHD increased linearly, according to the BLL
Hawari I 2020 [75]	Syria	Case-control	30, 29	3 to 12	10/20, 7/22	Hyperactivity	DSM-V, ADHD-RS	AAS	Blood	Pb ↔ (PH) BLLs were higher in the ADHD groups compared to the controls, but the difference was not statistically significant
Hong S 2015 [42]	South Korea	Case-control	1089	8 to 10 9.05	474/615	Combined ADHD, Inattention, Hyperactivity/Impulsivity	ADHD-RS (Parents and teachers reported)	GFAAS	Blood	Pb ↑ (PH) Pb ↔ (PI) BLLs showed a significant association with hyperactivity/impulsivity but not inattention
Huang S 2016 [43]	Mexico	Cross-sectional	412	6 to 13	NM	Combined ADHD, Inattention, Hyperactivity/Impulsivity	CRS-R	ICP-MS	Blood	Pb ↓ (PH) Pb ↔ (PI/PC) An association was observed between Pb exposure and hyperactivity/impulsivity when concurrent BLLs were ≤ 5 µg/dL, but not with inattention or combined subtypes
Ji Y 2018 [54]	USA	Cohort	1176, 299	9.6	681/499, 86/213	Combined ADHD	ICD-9, ICD-10	NM	Blood	Pb ↑ (PC) Pb levels ranging from 5–10 µg/dL significantly increase the odds of ADHD diagnosis by 66%
Joo H 2017 [68]	South Korea	Case-control	214, 214	7 to 12	61/153, 61/153	Combined ADHD, Inattention, Hyperactivity/Impulsivity	K-ARS (DSM)	AAS	Blood	Pb ↔ (PI/PH/PC) BLLs showed a significant association with all types of ADHD. However, after adjusting for postnatal secondhand smoke exposure, this association became non-significant
Kahn M 1995 [79]	USA	Case-control	85, 31	1 to 8	10/21,36/49	Combined ADHD	ADHD-RS (Parents reported), DSM-III	GFAAS	Blood	Pb ↔ (PC)
Kim J 2018 [13]	South Korea	Case-control	75, 75	6 to 17 9.8	28/47	Combined ADHD, Inattention, Hyperactivity/Impulsivity	K-SADS-PL, DSM-IV	GFAAS	Blood	Pb ↑ (PI/PH/PC) The ADHD group exhibited significantly elevated Pb concentrations compared to the health control group
Kim S 2013 [55]	USA	Case-control	58,71	5 to 12	23/35, 21/50	Combined ADHD	DSM-IV	ICP-MS	Blood	Pb ↑ (PC) An association was observed between BLLs (< 5 µg/dL) and ADHD.
Lee M 2018 [92]	Taiwan	Cross-sectional	46, PI: 29 PH: 47	6 to 16 8.1, PI: 8.0 P: 7.7	15/31, PI: 18/11 PH: 7/40	Inattention, Hyperactivity/Impulsivity	K-SADS-E, DSM-IV (Teachers reported)	ICP-MS	Urine	Pb ↑ (PI/PH) There was a positive correlation between Pb levels and inattention, hyperactivity/impulsivity
Li Y 2020 [91]	China	Case-control	106, 178	8.93, 8.30	53/53, 17/161	Combined ADHD	DSM-IV	ICP-MS	Urine	Pb ↑ (PC) The Pb level in the case group was 2.99 times higher compared to the control group
Lin Y 2019 [36]	China	Cross-sectional	164 High BLL: 88 Low BLL: 66 High bone lead level: 114 Low Bone Lead level: 46	3 to 15 Blood: 6.6, 6.3 Bone: 6.8, 6.3	Blood: 21/45, 27/61 Bone: 13/33, 36/78	Inattention, Hyperactivity/Impulsivity	VADPRS (Parents reported)	AAS	Blood	Pb ↔ (PI/PH/PC)
								KXRF	Bone	
Liu W 2014 [44]	China	Cross-sectional	Parents’ test: 117 Teachers’ test: 105	3 to 7 4.8	P: 31/86 T: 31/74	Combined ADHD, Hyperactivity/Impulsivity	ADHD symptom rating scale, DSM-IV	GFAAS	Blood	Pb ↑ (PH/PC) Child BLLs in the high (≥ 5 $\:\mu\:$g/dL) and low (< 5 $\:\mu\:$g/dL) Pb groups showed a positive correlation with impulsivity-hyperactivity and the ADHD
Luccchini R 2012 [82]	Italy	Cross-sectional	299	12.83	147/152	Inattention, Hyperactivity/Impulsivity, Combined ADHD	DSM-IV	GFAAS	Blood	Pb ↓ (PI/PH/PC) Weak borderline association between BLL and ADHD subscale
Maitre L 2021 [63]	UK, France, Lithuania, Spain, Norway, Greece	Cohort	1287	6 to 12	NM	Combined ADHD	CPRS, CBCL	Q-ICP-MS, AAS	Blood	Pb ↑ (PC) Childhood Pb exposure linked to increased ADHD index
Menezes-Filho J 2014 [74]	Brazil	Cross-sectional	70	7 to 12 9.5	36/34	Inattention	CBCL	GFAAS	Blood	Pb ↔ (PI)
Muñoz M 2020 [56]	Chile	Cross-sectional	All: 2500 ADHD: 170	3 to 17	36/134	Combined ADHD	Health records	AAS	Blood	Pb ↑ (PC) Children with Pb concentrations of ≥5 µg/dl have a 2.33-fold increased risk of developing ADHD
Namavar L 2018 [96]	Iran	Case-control	30,30	7	16/12, 12/18	Combined ADHD	CSI-4 (DSM-IV), CBCL	AAS	Nail	Pb ↑ (PC)
Nayak S 2023 [86]	India	Case-control	24,24	3 to 16 7.5, 8	NM	Combined ADHD, Inattention, Hyperactivity/Impulsivity	Psychiatrist/ Pediatrician	ICP-OES	Hair Urine	Pb ↑ (PC) ADHD cases had a median Pb level of 3.12 $\:\mu\:$g/g of hair, while controls had a median Pb level of 1.12 $\:\mu\:$g/g of hair Pb ↑ (PC)
Neugebauer J 2014 [35]	Germany	Cohort	114	6 to 10 9.5	NM	Combined ADHD, Inattention, Hyperactivity, Impulsivity	FBB-ADHS questionnaires	GC-HRMS	Maternal blood during pregnancy	Pb ↑ (PH/PC) The four ADHD scales in the FBB-ADHS demonstrated positive correlations with Pb, with a 20% increase in impulsivity observed for each doubling of Blood Pb concentrations. Additionally, the overall ADHD scale showed an approximate 9% increase per doubling of Pb concentration
Nigg J 2015 [39]	USA	Case-control	147, 122	6 to 17 12.5, 11.5	74/73, 39/83	Inattention, Hyperactivity, Impulsivity	ADHD-RS, CRS-R (DSM-IV)	ICP-MS	Blood	Pb (Teachers reported) ↑ (PI/PH) Pb (Parents reported) ↑ (PI/PH) BLL showed a correlation with scores derived from both teachers’ and parents’ reports assessing inattention and hyperactivity/impulsivity, even at population-typical BLLs
Nigg J 2008 [120]	USA	Case-control	53, 97	8 to 17 14.7, 12.5	21/32, 34/63	Combined ADHD, Inattention	CBCL (Parents and teachers reported), ADHD-RS, CRS-R, K-SADS-E (DSM-IV) (parents reported)	ICP-MS	Blood	Pb ↑ (PC) Pb ↔ (PI) The ADHD-C group showed elevated Pb levels compared to the control group, while the ADHD-PI group did not show any significant differences from the control group.
Nicolescu R 2010 [40]	Romania	Cross-sectional	83	8 to 12 9.9	41/42	Combined ADHD, Inattention, Hyperactivity/Impulsivity	CRS (ICD-10 and DSM-IV), FBB-ADHS (Parents and teachers reported)	ICP-MS	Blood	Pb (Teachers reported) ↑ (PI/PC) Pb (Parents reported) ↑ (PI/PH/PC)
Nigg J 2010 [58]	USA	Case-control	99, 108	6 to 17 11.8, 11.38	56/43, 31/77	Combined ADHD, Inattention	KSADS-PL (DSM-IV), ADHD Rating Scale (Teachers reported), CRS	ICP-MS	Blood	Pb ↑ (PC) Pb ↔ (PI) The combined group showed elevated Pb levels compared to the control group, while the inattention group did not display any significant differences from the control group
Park J 2016 [59]	South Korea	Case-control	114, 114	6 to 12 8.79, 8.73	27/87, 33/81	Combined ADHD, Inattention, Hyperactivity/Impulsivity	K-SADS-PL-K, ADHD-RS (DSM-IV) (Parents reported)	GFAAS	Blood	Pb ↑ (PC) Children with blood Pb concentrations exceeding 2.30 µg/dL were found to have a 2.5-fold higher risk of ADHD. Pb concentrations below 2.30 µg/dL did not show a significant effect.
Plusquellec P 2010 [41]	Canada	Cohort	110	4 to 6 5.4	56/44	Inattention, Impulsivity	IBRS (BSID-II,), behavioral coding of video recordings taken during fine motor testing	GFAAS	Cord Blood	Pb ↔ (PI/PH) No association was found between prenatal Pb exposure and child behavior
									Blood	Pb ↑ (PI/PH) Childhood Pb exposure is linked to increased impulsivity and observed inattention.
Perham J 2020 [89]	New Zealand	Case-control	52, 55	7 to 12 10.08, 9.78	0/52, 0/55	Combined ADHD, Inattention, Hyperactivity/Impulsivity	K-SADS-PL, ADHD Rating Scale-IV home (Parents reported)	ICP_MS	Hair	Pb ↔ (PI/PH/PC)
Renzetti S 2021 [29]	Italy	Cross-sectional	299	6 to 11 8.8	161/138	Combined ADHD, Inattention	CBCL	HR-ICP-MS	Blood	Pb ↔ (PI/PC)
Roy A 2009 [31]	India	Cross-sectional	756	3 to 7	352/404	Combined ADHD, Inattention, Hyperactivity	CADS (DSM-IV) (Teachers reported)	ASV	Blood	Pb ↑ (PI/PC) Pb ↔ (PH) BLLs were significantly associated with higher ADHD index Z-scores and inattention on the CADS-T assessment but not with hyperactivity.
Sioen I 2013 [121]	Belgium	Cohort	270	7 to 8 7.8	140/130	Hyperactivity	SDQ	HR-ICP-MS	Cord blood	Pb ↑ (PH) Prenatal Pb exposure showed a significant association with hyperactivity in children aged 7–8 years, with a doubling of exposure corresponding to an odds ratio of 3.43
Sears C 2022 [45]	USA	Cross-sectional	244	2 to 5 and 8	132/112	Hyperactivity	BASC-2	ICP-MS	Blood	Pb ↑ (PH) Elevated BLLs were associated with an increased likelihood of displaying at-risk or clinically significant hyperactivity.
Sehgal R 2020 [69]	India	Case-control	30, 30	6 to 12 9.1, 9.39	12/18, 0/30	Combined ADHD, Inattention, Hyperactivity/Impulsivity	DSM-IV, CPRS	ICP-AES	Blood	Pb ↔ (PI/PH/PC)
Setiawati Y 2019 [90]	Indonesia	Case-control	21, 23	6 to 12	6/15, 7/16	Combined ADHD	SRRS	AAS	Hair	Pb ↔ (PC)
Stein C 2022 [85]	USA	Cross-sectional	222	6 to 12 9.8	169/53	Combined ADHD, Inattention, Hyperactivity/Impulsivity	CADS	NM	Hair	Pb ↑ (PI/PC) ADHD-like behavior worsened with increasing Pb levels, as indicated by lower scores on Inattentive and Combined scales (adjusted for child age, sex, secondhand smoke exposure, HOME score, maternal education, and maternal IQ)
Skogheim T 2021 [97]	Norway	Nested case -control	1034, 705	12 to 19	329/705, 185/520	Combined ADHD	ICD-10	ICP-SFMS	Maternal Blood	Pb ↔ (PC)
Szkup-Jabłońska M 2012 [46]	Poland	Cross-sectional	78	2 to 18 8	16/62	Inattention, Hyperactivity/Impulsivity	ADHD-Rating Scale-IV (Parents reported)	GFAAS	Blood	Pb ↑ (PH) Pb ↔ (PI) High Pb levels correlate with hyperactivity/impulsiveness. No statistically significant correlations were found between children’s BLLs and ADD symptoms exhibited at home
Tabatadze T 2018 [33]	Georgia	Case-control	35, 35	6 to 8	17/18, 16/19	Combined ADHD	DSM V, ICD-10	Fluorescence spectrometry	Hair	Pb ↑ (PC)
Tuthill R 1996 [84]	USA	Cross-sectional	277	6.5 to 7.5	136/141	Combined ADHD	ABTR	ICP	Hair	Pb ↑ (PC) An association was found between physician-diagnosed ADHD and hair Pb levels in children. There is no apparent safe threshold for Pb
Viktorinova A 2016 [71]	Slovakia	Case-control	50, 58	6 to 14 8.9, 9.4	NM	Combined ADHD, Inattention, Hyperactivity	CAP (Teachers reported), CTRS, CPRS	ETA AAS	Blood	Pb ↔ (PI/PH/PC)
Wang H 2008 [32]	China	Case-control	630, 630	4 to 12	196/434, 196/434	Combined ADHD	K-SADS-E, DSM-IV-R (Parents and teachers reported)	ASV	Blood	Pb ↑ (PC) ADHD cases showed a significant association with elevated BLLs compared to lower BLLs across all sub-definitions
Wang Q 2009 [81]	China	Cross-sectional	317	6 to 12	154/163	Combined ADHD	CTRS	GFAAS	Blood Urine Hair Nail	Pb ↔ (PC) Children with blood Pb concentrations ≥ 100 µg/L had higher mean conner scores, but the difference was not statistically significant
Wang G 2003 [66]	China	Case-control	60, 60	NA	NA	Combined ADHD	DSM-IV	AAS	Blood	Pb ↑ (PC)
Winter A 2017 [48]	USA	Cohort	208	Age at blood test: younger than 6 Impulsivity test: 16–18	111/97	Hyperactivity/Impulsivity	CBCL	NM	Blood	Pb ↑ (PH)
Xu Y 2015 [61]	China	Case-control	50, 50	4 to 12 8.3, 8.1	10/40, 10/40	Combined ADHD	K-SADS-E, DSM-IV-R	NM	Blood	Pb ↑ (PC) 14.0% of healthy children had Pb concentration < 6 µg/dL, compared to 3.9% in ADHD patients. Higher Pb concentrations > 15 µg/dL were consistently more prevalent in the ADHD group. ADHD patients also exhibited significantly larger BLLs than controls
Yu C 2016 [80]	Taiwan	Case-control	105, 46	4 to 15 8.9, 9.2	68/91, 25/148	Combined ADHD	DSM-IV (Teacher reported)	ICP-MS	Blood	Pb ↔ (PC) No significant difference in BLLs was observed in children with and without ADHD
Yousef S 2011 [60]	UAE	Case-control	74, 18	5 to 15 8.3	NM	Combined ADHD, Inattentive, Hyperactivity	DSM-IV	ICP-MS	Blood	Pb ↑ (PC) Significantly higher BLLs were observed in the ADHD group compared to controls. ADHD odds ratio increased by 5.2% per 1 ppb increase in Pb concentration
Yang R 2019 [70]	China	Case-control	395, 419	6 to 16 8.9, 8.8	35/360, 33/386	Combined ADHD, Inattention, Hyperactivity/Impulsivity	DSM-V	GFAAS	Blood	Pb ↔ (PI/PH/PC)
Vafaee-Shahi M 2022 [62]	Iran	Case-control	50, 30	5 to 12 7.6, 8.1	21/29, 13/17	Combined ADHD	DSM-IV	AAS	Blood	Pb ↑ (PC) The Blood Pb mean in ADHD children significantly exceeded the normal group
Zhang R 2015 [14]	China	Cross-sectional	243	3 to 7 5.1	102/141	Combined ADHD, Inattention, Hyperactivity/Impulsivity	C-ARS (DSM) (Parents reported)	GFAAS	Blood	Pb ↑ (PI/PH/PC) Children with high BLLs had a 2.4-fold increased risk of ADHD compared to those with low BLLs

*NM* Not Mentioned, *ND* Not Detected, *ADHD* Attention-Deficit Hyperactivity Disorder, *ADD* Attention-Deficit Disorder, *F/M* Female/Male, *PC* predominantly Combined, *PI* predominantly Inattention, *PH* predominantly Hyperactive, *DSM* Diagnostic and Statistical Manual Of Mental Disorders, *ICP-MS* Inductively Coupled Plasma Mass Spectrometry, *AAS* Atomic Absorption Spectrometry, *ICP-OES* Inductively Coupled Plasma -Optical Emission Spectroscopy, *VADRS* Vanderbilt ADHD Diagnostic Rating Scale, *BASC-2* The Behavior Assessment System for Children Second Edition, *ICD* The International Classification Of Diseases, *K-SADS-PL* Kiddie-Schedule for Affective Disorders and Schizophrenia Present and Lifetime Version, *VADPRS* Vanderbilt ADHD Diagnostic Parent Rating Scale, *SDQ* The Strengths and Difficulties Questionnaire,, *NHANES Dataset* The National Health And Nutrition Examination Survey Dataset, *CPRS* Conners’ Parents Rating Scale, *CRS-R* The Conners Rating Scales-Revised, *IBRS* Infant Behavior Rating Scale, *BSID-II* The Bayley Scales of Infant and Toddler Development Second Edition, *DBD* The Disruptive Behavior Disorders Rating Scale, *CTRS* Conners’ Teacher Rating Scale, *CBCL* Child Behavior Checklist, FBB-ADHS questionnaires: German ADHD Rating Scale, *CV-AAS* Cold Vapor Atomic Absorption Spectrometry, *DMA* Direct Mercury Analyzer, *CRS* Conners’ Rating Scale, *DBDRS* Disruptive Behavior Disorder Rating Scale, *TDA-AAS* Thermal Decomposition Amalgamation Atomic Absorption Spectrometry, *HBV* Hepatitis B Virus, *HiB* Hemophilus Influenzae Type B, *DTaP* Diphtheria Tetanus Pertussis, *DTP* Diphtheria Tetanus Pertussis, *DT* Diphtheria Tetanus, *USA* United States of America, *UK* United Kingdom

**Table 3:** Characteristics of included studies for assessments of the relationship between mercury concentrations in different biological samples and ADHD

First author (year)	Country	Type of Study	Sample size (control, case)	Mean age (control, case)	Gender F/M (control, case)	Diagnostic criteria for ADHD	ADHD Symptoms Measured	Assessment method	Exposure Measurement	Main result
Al-Ayadhi L 2005 [88]	Saudi Arabia	Case-control	80, 8	Up to 14 7.2, 6	NM, 0/8	E-2	Inattention	AAS	Hair	Hg ↔ (PI)
Almotawah F 2019 [34]	Saudi Arabia	Cross-sectional	667, 202 M: 796	6 to 12	252/415, 105/97	VADPRS	Combined ADHD	Fluorescence spectrometry	Child saliva	Hg ↑ (PC) Higher Hg levels associated with increased risk of ADHD
Andrews N 2004 [106]	UK	Cohort	222	3.7	51/171	ICD-9	Inattention	doses of DTP/DT	Maternal saliva	Hg ↑ (PC) Prenatal Hg exposure linked to increased risk of ADHD behavior
Barry M 2020 [100]	Saudi Arabia	Case-control	(90,90)	6 to 16	43/47, 43/47	VADRS, Attending a special school for ADHD children	Combined ADHD	CV-AAS	Saliva	Hg ↑ (6–7 y/o) (PC) Hg ↔ (12–16 y/o) (PC) ADHD children had elevated salivary Hg levels compared to non-ADHD children, specifically in the 6–7 years age group
Boucher O 2012 [49]	Canada	Prospective longitudinal study	279	11.3	141/138	DBD (Teachers reported) (DSM-IV)	Inattention, Hyperactivity/Impulsivity	ICP-MS	Blood	Hg ↔ (PI/PH)
Chan T 2015 [95]	USA	Cross-sectional	262	11 to 13	128/138	DBDRS	ADHD, Inattention, Hyperactivity/Impulsivity	CV-AAS	Cord blood	Hg ↑ (PI) Hg ↔ (PH)
Dikme G 2013 [78]	Turkey	Case-control	59,17	1.6 to 16	NM	DSM-IV	Combined ADHD	AAS	Blood	Hg ↔ (PC)
Ethier A 2015 [73]	Canada	Cohort	27	8.6 to 12.6 11.2	9/18	Classic Posner paradigm	Inattention, Impulsivity	CVAAS	Blood	Hg ↔ (PI/PH)
Gari M 2022 [23]	Poland	Cohort	436	Parental age: 29.3	NM	SDQ	Hyperactivity, Inattention	CV-AAS	Cord blood	Hg ↔ (PI/PH)
Geier D 2014 [122]	USA	Cohort	20584, 1485	5.7, 5.7	10281/10303, 327/1158	ICD-9	Combined ADHD	HBV vaccine exposure	--	Hg ↑ (PC) Hyperkinetic syndrome of childhood cases had higher organic-Hg exposure compared to controls, per µg basis
Geier D 2017 [105]	USA	Cohort	9997, 1041	5.7, 5.7	820/5039, 221/4958	ICD-9	Combined ADHD, Inattention	HiB vaccine exposure	--	Hg ↑ Cases diagnosed with ADD/ADHD had significantly higher Hg exposure than controls on a per 25 µg Hg basis
Geier D 2005 [103]	USA	Cohort	374	49 months	75/299	ICD-9	Inattention	HBV, HiB, DTaP vaccine exposure	--	Hg ↑ Significant positive correlations were found (without adjusting for multiple comparisons) at 1 µg exposure for ADD
Geier D 2018 [104]	USA	Cross-sectional	4185, 208	16.02, 13	2097/2088, 97/111	The NHANES Dataset	Combined ADHD	HBV vaccine exposure	--	Hg ↑ Infant Thimerosal-containing hepatitis B vaccine exposure significantly increased ADHD risk
Ha M 2009 [53]	South Korea	Cohort	1663, 115	6 to 10	NM	CPRS	Combined ADHD	CV-AAS	Blood	Hg ↔ (PC)
Kim S 2013 [55]	USA	Case-control	58,71	5 to 12	23/35, 21/50	DSM-IV	Combined ADHD	ICP-MS	Blood	Hg ↔ (PC)
Lee M 2018 [92]	Taiwan	Cross-sectional	46, PI: 29 PH: 47	6 to 16 8.1, PI: 8.0 PH: 7.7	15/31, PI: 18/11 PH: 7/40	K-SADS-E, DSM-IV (Teachers reported)	Inattention, Hyperactivity/Impulsivity	ICP-MS	Urine	Hg (Parents reported) ↑ (PH) Hg (Parents reported) ↔ (PI) Hg (Teachers reported) ↔ (PI/PH) Hg levels correlated positively with parent-rated hyperactivity scores
Lin P 2018 [101]	Taiwan	Cohort	44034, 44034	Younger than 20 9.58, 9.56	22238/21796, 22238/21796	ICD-9	Combined ADHD	--	Teeth	Hg ↔ (PC) Amalgam restorations in young patients are not associated with increased ADHD risk.
Lozano M 2020 [28]	Spain	Cohort	385	9 to 11 years	NM	CPRS-R	Combined ADHD, Hyperactivity/Impulsivity	DMA	Hair	Hg ↔ (PC) Children’s total Hg concentrations showed a positive linear association with the ADHD index, but the relationship was not statistically significant
Lygre G 2018 [102]	Norway	Cohort	At 3: 42163 at 5: 23302	3 and 5	NM	CBCL, DSM-IV(Teachers reported)	Combined ADHD	--	Maternal amalgam filling	Hg ↔ (PC) No significant associations were found between teeth with amalgam filling/removal during pregnancy and ADHD symptoms in 3-5-year-old children.
Nicolescu R 2010 [40]	Romania	Cross-sectional	83	8 to 12 9.9	41/42	CRS (ICD-10 and DSM-IV), FBB-ADHS (Parents and teachers reported)	Combined ADHD, Inattention, Hyperactivity/Impulsivity	ICP-MS	Blood	Hg (Parents and teachers reported) ↔ (PI/PH/PC)
Patel N 2019 [99]	USA	Cohort	320	13 to19	175/145	BASC-2 (Parents reported)	Inattention, Hyperactivity/Impulsivity	ICP-MS	Maternal Blood	Hg ↔ (PI/PH)
Perham J 2020 [89]	New Zealand	Case-control	52, 55	7 to 12 10.08, 9.78	0/52, 0/55	K-SADS-PL, ADHD Rating Scale-IV home (Parents reported)	Combined ADHD, Inattention, Hyperactivity/Impulsivity	ICP_MS	Childhood Blood	Hg ↔ (PI/PH)
Plusquellec P 2010 [41]	Canada	Cohort	110	4 to 6 5.4	56/44	IBRS (BSID-II), Behavioral coding of video recordings taken during fine motor testing	Inattention, Impulsivity	AAS	Cord Blood	Hg ↔ (PI/PH)
Renzetti S 2021 [29]	Italy	Cross-sectional	299	6 to 11 8.8	161/138	CBCL	Combined ADHD, Inattention	TDA-AAS DMA-80	Blood	Hg ↔ (PI/PH)
Sehgal R 2020 [69]	India	Case-control	30, 30	6 to 12 9.1, 9.39	12/18, 0/30	DSM-IV, CPRS	Combined ADHD, Inattention, Hyperactivity/Impulsivity	ICP-AES	Blood	Hg ↑ (PH) Hg ↔ (PI/PC) Blood mercury showed a significant correlation with the hyperactivity-impulsivity T score.
Skogheim T 2021 [97]	Norway	Nested case -control	1034, 705	12 to 19	329/705, 185/520	ICD-10	Combined ADHD	ICP-SFMS	Maternal Blood	Hg ↓ (PC) Gestational Hg is linked to reduced child ADHD risk
Sagiv S 2012 [30]	USA	Cohort	421	8.2	209/212	CTRS (DSM-IV)	Combined ADHD, Inattention, Hyperactivity/impulsivity	DMA-80	Maternal Hair	Hg ↑ (PI/PH/PC) A 1 microg/g threshold for Hg levels was observed about CTRS. Piecewise regression analysis indicated a protective association between Hg levels and inattentive, impulsive/hyperactive, and combined behaviors when Hg levels were below 1 µg/g. However, an increase in the risk of these behaviors was found when Hg levels reached or exceeded 1 µg/g, especially for impulsivity/hyperactivity
Tabatadze T 2018 [33]	Georgia	Case-control	35, 35	6 to 8	17/18, 16/19	DSM-V, ICD-10	Combined ADHD	Fluorescence spectrometry	Hair	Hg ↑(PC)
Yousef S 2011 [60]	UAE	Case-control	74, 18	5 to 15 8.3	NM	DSM-IV	Combined ADHD, Inattentive, Hyperactivity	ICP-MS	Blood	Hg ↔ (PC)

*NM* Not Mentioned, *ND* Not Detected, *ADHD* Attention-Deficit Hyperactivity Disorder, *ADD* Attention-Deficit Disorder, *F/M* Female/Male, *PC* predominantly Combined, *PI* predominantly Inattention, *PH* predominantly Hyperactive, *DSM* Diagnostic and Statistical Manual Of Mental Disorders, *ICP-MS* Inductively Coupled Plasma Mass Spectrometry, *AAS* Atomic Absorption Spectrometry, *ICP-OES* Inductively Coupled Plasma -Optical Emission Spectroscopy, *VADRS* Vanderbilt ADHD Diagnostic Rating Scale, *BASC-2* The Behavior Assessment System for Children Second Edition, *ICD* The International Classification Of Diseases, *K-SADS-PL* Kiddie-Schedule for Affective Disorders and Schizophrenia Present and Lifetime Version, *VADPRS* Vanderbilt ADHD Diagnostic Parent Rating Scale, *SDQ* The Strengths and Difficulties Questionnaire,, *NHANES Dataset* The National Health And Nutrition Examination Survey Dataset, *CPRS* Conners’ Parents Rating Scale, *CRS-R* The Conners Rating Scales-Revised, *IBRS* Infant Behavior Rating Scale, *BSID-II* The Bayley Scales of Infant and Toddler Development Second Edition, *DBD* The Disruptive Behavior Disorders Rating Scale, *CTRS* Conners’ Teacher Rating Scale, *CBCL* Child Behavior Checklist, FBB-ADHS questionnaires: German ADHD Rating Scale, *CV-AAS* Cold Vapor Atomic Absorption Spectrometry, *DMA* Direct Mercury Analyzer, *CRS* Conners’ Rating Scale, *DBDRS* Disruptive Behavior Disorder Rating Scale, *TDA-AAS* Thermal Decomposition Amalgamation Atomic Absorption Spectrometry, *HBV* Hepatitis B Virus, *HiB* Hemophilus Influenzae Type B, *DTaP* Diphtheria Tetanus Pertussis, *DTP* Diphtheria Tetanus Pertussis, *DT* Diphtheria Tetanus, *USA* United States of America, *UK* United Kingdom

### Lead (pb)

#### Blood

A total of seventy-four studies examined the link between Lead and Attention-Deficit Hyperactivity Disorder (ADHD). Fifty-four studies measured whole blood lead concentrations [12–14, 29, 31, 32, 36–82] between 1988 and 2022. Regardless of the ADHD subtype, a total of thirty-four studies found an association between increased lead levels and ADHD occurrences (14 case-control studies, 11 cross-sectional studies, and 9 cohort studies). Twenty-four studies reported that children with a combined ADHD subtype had higher blood lead levels [12–14, 31, 32, 40, 44, 50–66], while fourteen studies reported non-significant results (9 case-control studies, and five cross-sectional studies) [14, 29, 36, 43, 67–69, 71, 72, 77–81]. The Inattention subtype of ADHD was positively associated with blood lead levels in nine studies [12–14, 31, 37–41]. In contrast, nineteen studies found no significant association between the inattention subtype and blood lead levels (9 case-control studies, seven cross-sectional studies, and 3 cohort studies) [29, 36, 37, 42, 43, 46, 47, 49, 50, 57, 58, 67–74]. The increasing impact of blood lead concentrations on ADHD hyperactivity was documented in thirteen studies [13, 14, 37, 39–42, 44–49], while thirteen reported non-significant results (6 case-control studies, five cross-sectional studies, and 2 cohort studies) [14, 31, 36, 37, 50, 67–69, 71–73, 75, 76]. Conversely, Lucchini et al. (2012) reported that all three subtypes of ADHD are associated with lower blood lead levels [82]. Furthermore, Huang et al. (2016) found a positive correlation between lower blood lead levels and an increased risk of the hyperactivity subtype in children with ADHD [43]. Table 2 shows the findings of these studies in alphabetical order.

#### Hair

Ten studies were conducted to assess lead concentrations in the hair of children with ADHD. Seven types of research found elevated Pb levels in hair associated with ADHD between 1998 and 2023, regardless of subtype (5 case-control studies and two cross-sectional studies) [33, 83–88]. Six studies found an association between hair Pb levels and Combined ADHD subtype [33, 83–87], While no significant result was found between the three studies [81, 89, 90]. In three research studies, higher hair Pb levels have been linked to inattention ADHD [83, 85, 88]. One reported no significant association between the hair Pb levels and the inattention subtype [89]. There is a positive correlation between higher hair Pb levels and the hyperactivity subtype of ADHD, according to Amgalan et al. [83], whereas Perham et al. (2020) did not find a significant association [89]. Table 2 shows details of these studies in alphabetical order.

#### Urine

Pb levels in urine were measured in six studies between 1983 and 2023. Two studies reported elevated Pb levels in urine are associated with combined ADHD (2 cross-sectional studies) [86, 91]. Wang et al. (2019) did not find any significant association [81]. According to Lee (2018), there is a positive correlation between the inattention and hyperactivity subtypes of ADHD and Urinary Pb levels [92], while Gittelman et al. (1983) found no significant correlation [93]. Table 2 shows the findings of these studies in alphabetical order.

#### Teeth

A total of two studies measured lead concentrations in teeth, but only one found an association between higher levels of Pb and inattention and hyperactivity subtypes of ADHD (a cohort study) [94]. In contrast, the other found no relation with any of the three subtypes [95]. Table 2 presents the results of these studies listed in alphabetical order.

#### Nail

Lead concentration in nails was measured in two studies. One reported that higher Pb nail levels are correlated with the combined ADHD subtype (a case-control study) [96]. In contrast, the other did not report significant results (a cross-sectional study) [81]. Table 2 shows the findings of these studies in alphabetical order.

#### Bone

Lin et al. (2017) measured Pb concentrations in bone but found no significant correlation between bone Pb levels and ADHD [36]. Table 2 shows the findings of this study in alphabetical order.

#### Cord blood

Seven studies measured the level of lead in cord blood. Fraser et al. (2006) could not detect Pb levels in cord blood [47]. In four studies, Pb levels in cord blood were unrelated to the hyperactivity subtype of ADHD (4 cohort studies). In contrast, in two studies, they were positively correlated (1 cross-sectional study and 1 cohort study). Three studies found no significant association between cord blood Pb levels and ADHD inattention. As opposed to that, Ethier (2015) discovered that high cord blood Pb levels are associated with inattention ADHD [73]. Table 2 presents the results of these studies in alphabetical sequence.

#### Maternal blood

A lead level was measured in the blood of mothers of ADHD children in two studies. Neugebauer found that greater Pb levels in maternal blood increase the risk of hyperactivity and combined ADHD [35], whereas Skogheim (2021) did not report any significant association between maternal blood Pb levels and combined ADHD [97]. Table 2 shows the findings of these studies in alphabetical order.

#### Maternal urine

The lead level in the urine of ADHD children’s mothers has been examined in one study. However, no significant correlation has been found [98]. Table 2 shows the findings of this study in alphabetical order.

### Mercury (hg)

#### Blood

The relationship between Mercury level and attention deficit hyperactivity disorder was examined in twenty-nine studies between 2009 and 2021. Mercury blood levels in children with ADHD were examined in ten studies. Six reported non-significant results between blood Hg level and combined ADHD (4 case-control studies, one cross-sectional study, and 1 cohort study) [40, 53, 55, 60, 69, 78]. Six found no correlation between Hg level and Inattention ADHD (1 case-control study, one cross-sectional study, and 4 cohort studies) [40, 41, 49, 69, 73, 99]. Five more studies found no link between blood Hg level and hyperactive subtype (1 cross-sectional study and 4 cohort studies) [40, 41, 49, 73, 99]. In contrast, only Sehgal (2020) discovered a link between blood Hg level and hyperactivity subtype [69]. Table 3 presents the results of these studies listed in alphabetical order.

#### Hair

Five studies assessed the Mercury level in the hair between 2012 and 2020. Tabatadze et al. (2018) discovered that increased hair Hg levels are connected with the combined subtype of ADHD [33]. However, three other studies showed no significant connection (one case-control study, one cross-sectional study, and one cohort study) [28, 29, 89]. Three studies found no conclusive link between hair Hg levels and the inattention subtype (two case-control studies and one cross-sectional study) [29, 88, 89]. Table 3 shows the findings of these studies in alphabetical order.

#### Saliva

Two studies examined the quantity of mercury in saliva. Both studies revealed a link between higher Hg levels in saliva and children with comorbid ADHD [34, 100]. Table 3 shows more details of these studies in alphabetical order.

#### Teeth

Mercury levels in teeth were measured in two studies. Hg level in teeth could not be detected by Chan [95]. Additionally, Lin and colleagues (2017) did not discover a connection between combined ADHD and teeth Hg level [101]. Table 3 shows the findings of these studies in alphabetical order.

#### Urine

Lee et al. (2018) measured the mercury level in urine [92]. Furthermore, there was a significant connection between elevated urine Hg level and the Hyperactivity subtype of ADHD, but not with the Inattention subtype [92]. Table 3 shows details of this study in alphabetical order.

#### Maternal hair

Two studies assessed the mercury content of the mothers’ hair of ADHD children. Additionally, both studies found higher amounts of Hg in the maternal hair of ADHD offspring (2 cohort studies), which is associated with all subtypes of the disorder [23, 30]. The table presents the results of these studies, which are listed alphabetically.

#### Maternal blood

The Mercury level in maternal blood was measured in two studies. One did not find any significant correlation between maternal blood Hg level and hyperactivity/inattention ADHD subtypes [99]. Whereas, Skogheim et al. (2021) reported that decreased Hg levels in maternal blood are related to combined ADHD [97]. Table 3 shows the findings of these studies in alphabetical order.

#### Maternal saliva

One study looked into the association between mercury concentration in maternal saliva and child ADHD and concluded that higher Hg levels are linked to the combined subtype of ADHD [34]. Another study examined the link between maternal amalgam filling and child ADHD but found no significant results [102]. Table 3 shows the findings of these studies in alphabetical order.

#### Cord blood

The Mercury level in cord blood was measured in three studies. Two studies did not discover any significant result [41, 73], while Boucher 2012 found a correlation between elevated cord blood Hg level and inattention ADHD [49]. Table 3 shows the findings of these studies in alphabetical order.

#### Vaccination

Five studies were conducted to investigate the link between Thimerosal vaccination exposure and ADHD. Four of them found that a higher vaccine dosage is linked to ADHD [103–105]. In contrast, Andrews discovered a decreasing trend in ADHD by immunization dosage in 2004 [106]. Table 3 shows details of these studies in alphabetical order.

## Discussion

The outcomes of this systematic review reveal a substantial correlation between lead exposure and ADHD, as evidenced by nearly two-thirds of the seventy-four studies that examined lead levels in various biological samples being associated with at least one of the ADHD subtypes.

In our systematic review, we took a more comprehensive approach by encompassing a broader range of literature published from 1983 to 2023. Our analysis expanded to include more diverse biological samples, including blood, urine, nails, hair, and teeth. By doing so, we aimed to enhance the overall comprehensiveness of our investigation into the association between lead exposure and ADHD in children. Also, we include studies on maternal lead levels and the occurrence of ADHD in their children. Our systematic review findings were mixed regarding the maternal and cord blood lead levels and the occurrence of ADHD, which underscores more studies in this field. According to scientific investigation, it has been firmly established that lead can cross the placental barrier and enter the fetal circulation as early as the 12th week of gestation, maintaining its presence throughout the entirety of the developmental process until birth [107, 108].

The human body can be exposed to lead through various pathways, including ingesting contaminated food, water consumption from contaminated supply systems, contact with lead-based paint, exposure to secondhand smoke, and inhaling air pollutants. Children are especially susceptible to lead poisoning [19]. Lead contamination in food is the primary source of nonoccupational lead exposure, originating from diverse sources encompassing soil, air, and water pollutants and agricultural processes throughout various stages, such as harvesting, processing, packaging, and preparation [19, 109]. Passive tobacco smoking represents a significant source of lead exposure. In a study conducted by Serdar et al. [110], it was observed that children living in households with smokers had hair lead levels that were more than double those of children in households without smokers. Children who play with toys are at a high risk of lead exposure, particularly from PVC toys, which contain lead as a component. This risk is further exacerbated when the toys are coated with lead-based paints. The issue becomes more severe when children habitually chew, suck, or lick these toys, leading to the ingestion of significant amounts of lead [111]. In addition to the ways mentioned above, leaded gasoline was previously identified as an important source of lead exposure. However, removing leaded gasoline has reduced airborne lead pollutants [112]. Nevertheless, the amount of time spent in.

Our findings indicate that most included studies reported no association between pre and postnatal mercury exposure and any ADHD symptoms. However, it is important to note that the available evidence on the impact of prenatal and postnatal mercury exposure on the prevalence of ADHD is limited. Due to this limitation and the heterogenicity of the studies, it is challenging to reach any conclusive findings or draw definite conclusions from the results. These findings are consistent with the study conducted by Tapia et al. in 2023, which examined the correlation between mercury exposure and neurodevelopmental diseases among children [27].

There are several sources of mercury exposure, particularly methylmercury, the most hazardous form of Hg. The primary source for human populations is fish consumption.

In the past, mercury exposure posed a significant concern due to the widespread use of mercury dental amalgam fillings. However, these have now been replaced by alternative materials [113]. A study conducted by Ulukapi analyzed mercury levels in the urine of individuals with amalgam fillings and found that their levels fell within the normal range [114]. It is important to note that the mercury concentration in the air is generally low and does not pose a significant risk to human health [22]. Currently, the main concern regarding mercury exposure stems from the discharge of mercury into waterways by industries and occupational exposure [115].

Our study reveals that exposure to mercury through the preservative Thimerosal poses a risk factor for the diagnosis of ADHD. Thimerosal contains ethylmercury and has historically been included in various vaccines since the 1930s. It is still used in several childhood vaccines, including tetanus toxoid, Hib, HBV, DTP, DT, and influenza [116]. Ethylmercury, produced when Thimerosal-containing vaccines break down, can traverse the BBB. However, the half-life of ethylmercury is shorter, leading to lower peak concentrations in the blood upon repeated exposure [117]. Although studies on the toxicity of Thimerosal in the human population are limited, existing research has indicated no notable differences in toxicity between methylmercury and ethylmercury. It has been demonstrated that the accumulation of Hg^2+^ in the brain is greater following exposure to ethylmercury than methylmercury exposure [22].

## Limitations

Our study’s literature review revealed some potential limitations. A significant limitation is that many studies relied on questionnaires filled out by parents or teachers to diagnose ADHD, which could introduce the risk of misdiagnosis or biases. A more appropriate approach to reduce this risk and improve diagnostic accuracy would have been for physicians to use a medical diagnosis of ADHD based on established diagnostic criteria, such as the ICD or DSM, thereby decreasing the likelihood of misdiagnosis. Additionally, various biological materials, including blood, hair, urine, teeth, and bone, have been analyzed by researchers in this particular field. There may be notable variations in the outcomes observed across different laboratories utilizing distinct techniques. Consequently, interpreting these findings can present a challenge due to the biological samples’ inherent characteristics. Specifically, the distribution of elements within a tooth is not uniform, and their levels differ depending on the type of tooth, which correlates with its age [118]. Urine cannot reflect long-term metal exposure either [98].

Additionally, it is important to highlight that the studies examined in our review employed varying observation and exposure times, which needed to be more consistent across all research investigations. These studies also encompassed different age groups, adding to the heterogeneity of the findings. This review included studies spanning several decades; we observed consistent findings on metal levels’ effects across the older and more recent publications. Future longitudinal analyses examining the potential impact of evolving environmental regulations and industrial practices on metal exposures could provide valuable insights into the temporal trends of these contaminants and their relationship with ADHD.

Variations in methodologies and the considerable heterogeneity within the literature should be considered when interpreting our findings. Also, studies did not report the concentration of these metals in their studies, and the lack of numerical data prevented us from executing a meta-analysis on this matter. Another notable issue is that studies should have mentioned the isotope of Hg and Pb in which they have been measured. Therefore, we could not organize the studies using their isotope.

## Data Availability

No datasets were generated or analysed during the current study.
