# Correction: Exploring the link between toxic metal exposure and ADHD: a systematic review of pb and hg

**DOI:** 10.1186/s11689-026-09741-w

**Published:** 2026-10-27

**Authors:** Reyhane Farmani, Omid Mehrpour, Alireza Kooshki, Samaneh Nakhaee

**Affiliations:** 1 Student Research Committee, Birjand University of Medical Sciences, Birjand, Iran; 2 Michigan Poison & Drug Information Center, Wayne State University School of Medicine, Detroit, MI USA; 3 Medical Toxicology and Drug Abuse Research Center (MTDRC), Birjand University of Medical Sciences, Birjand, Iran

**Correction: Journal of Neurodevelopmental Disorders (2024) 16:44**

**https://doi.org/10.1186/s11689-024-09555-8**

In this article, in the “Methods – Design and search strategy” section, the final sentence, “The study has been registered in PROSPERO with ID number 557,671,” should have been removed.

In Table 1 of this article (Search strategies in different databases for retrieving the relevant documents), in the Google Scholar row, the Results value has been corrected from 222 to 209.

The original article has been corrected.

